# The Dual Role of cGAS-STING Signaling in COVID-19: Implications for Therapy

**DOI:** 10.3390/cells14050362

**Published:** 2025-02-28

**Authors:** Daniele Castro di Flora, João Paulo Zanardini Lara, Aline Dionizio, Marília Afonso Rabelo Buzalaf

**Affiliations:** Department of Biological Sciences, Bauru School of Dentistry, University of São Paulo, Bauru 17012-901, Brazil; dani_diflora@yahoo.com.br (D.C.d.F.); joaopaulolara95@gmail.com (J.P.Z.L.); alinesdionizio@usp.br (A.D.)

**Keywords:** SARS-CoV-2 infection, COVID-19, cGAS–STING pathway, innate immunity

## Abstract

The progression of COVID-19 involves a sophisticated and intricate interplay between the SARS-CoV-2 virus and the host’s immune response. The immune system employs both innate and adaptive mechanisms to combat infection. Innate immunity initiates the release of interferons (IFNs) and pro-inflammatory cytokines, while the adaptive immune response involves CD4+ Th lymphocytes, B lymphocytes, and CD8+ Tc cells. Pattern recognition receptors (PRRs) recognize pathogen-associated molecular patterns (PAMPS) and damage-associated molecular patterns (DAMPs), activating the cyclic guanosine monophosphate-adenosine monophosphate synthase-stimulator of interferon genes (cGAS-STING) signaling pathway, a crucial component of the innate immune response to SARS-CoV-2. This pathway fulfills a dual function during infection. In the early phase of infection, the virus can suppress cGAS-STING signaling to avoid immune detection. However, in the late stages, the activation of this pathway may trigger excessive inflammation and tissue damage, exacerbating disease severity. Modulating the cGAS-STING pathway, whether through agonists like dimeric amidobenzimidazole (diABZI) or inhibitors targeting viral proteins, such as 3CLpro, for example, offers a promising approach for personalized therapy to control the immune response and mitigate severe inflammation, ultimately improving clinical outcomes in patients with severe COVID-19.

## 1. Introduction

Since late 2019, coronavirus disease 2019 (COVID-19) has rapidly spread across the globe, resulting in nearly 800 million confirmed cases and 7 million deaths to date. The COVID-19 pandemic has affected the entire world with a profound impact on public health and the global economy (https://www.who.int, accessed on 4 December 2024). The pathogenesis of the disease is driven by the interaction between the severe acute respiratory syndrome coronavirus 2 (SARS-CoV-2) infection and dissemination and the body’s attempt to eliminate the virus. This leads to direct and indirect cellular damage and dysfunction.

SARS-CoV-2 is a β-coronavirus in which the genome is constituted by 14 open reading frames (ORFs) encoding structural proteins (membrane protein M, spike protein S, nucleocapsid protein N, and envelope protein E), 16 nonstructural proteins (nsp), and 9 accessory proteins (ORF3a, ORF3b, ORF6, ORF7a, ORF7b, ORF8, ORF9b, ORF9c, and ORF10). Among them, spikes are essential for the infection mechanism, as they interact with ACE2 (angiotensin-converting enzyme 2) on host cells, facilitating viral entry [1].

Once inside the host cells, the virus’s interaction with the host’s immune system drives the course of the disease. The immune system consists of both innate and acquired defense mechanisms. The innate response triggers infected cells to release interferons (IFNs) and pro-inflammatory cytokines. IFNs induce non-infected cells to enter an antiviral state, while pro-inflammatory cytokines mobilize macrophages and other phagocytes to eliminate viruses and infected cells. The adaptive immune responses involve CD4+ Th lymphocytes, which are activated by cytokines and subsequently stimulate B-lymphocytes to generate neutralizing antibodies to combat the virus and CD8+ Tc cells to induce programmed death in infected cells. Essentially, the innate response identifies the virus and coordinates the adaptive immune response to combat the infection. The effectiveness of these responses hinges on their timely coordination. A swift production of IFNs within 18 to 24 h after infection results in well-coordinated innate and acquired immune responses. However, if IFN production is delayed (typically 3 to 4 days after infection), both immune responses become ineffective [2]. This delay occurs despite the persistent release of IL-6 and TNF-α by various cells, the infiltration of monocytes, and inflammatory reactions. Additionally, a dysfunctional immune response is further exacerbated by macrophages, potentially culminating in the severe form of COVID-19 [3]. In such cases, there is a significant upsurge in cytokine release, a phenomenon known as cytokine release syndrome (CRS), also referred to as a “cytokine storm”.

The innate immune system acts as the organism’s initial line of defense against invading pathogens [4]. It consists of both constitutive and inducible mechanisms to eliminate infection. The constitutive immune mechanisms include the body’s chemical and physical barriers, such as saliva, skin, stomach acid, and urine flow. Although these barriers provide an immediate response, they lack the ability to amplify the immune reaction. This amplification is carried out provided by the inducible mechanisms, which function through pattern recognition receptors (PRRs), such as Nod-like receptors (NLRs), Toll-like receptors (TLRs), RIG-I-like receptors (RLRs), and the DNA sensor cyclic guanosine monophosphate (GMP)-adenosine monophosphate (AMP) synthase (cGAS)-stimulator of interferon genes (STING) signaling pathway. These receptors enable the innate immune system to identify both pathogen- and damage-associated molecular patterns (PAMPs/DAMPs). Among these receptors, the cGAS-STING pathway plays a critical role in the innate response to infections. Mechanistically, when self or pathogen double-stranded DNA (dsDNA) accumulates in the cytoplasm, it activates cGAS, leading to the production of cyclic GMP-AMP (cGAMP). This molecule subsequently binds to and activates STING [5,6,7]. Once activated, STING then relocates from the endoplasmic reticulum (ER) to the Golgi apparatus, where it recruits the TANK-binding kinase 1 (TBK1) and stimulates IkB kinase (IKK). This process results in the phosphorylation of interferon regulatory factor 3 (IRF3) and nuclear factor-kB (NF-kB) [8,9]. As a result, the transcription of type I interferons (IFNs) and other inflammatory genes is initiated, facilitating the immune response to eliminate pathogens [10,11,12,13].

Within the host cells, SARS-CoV-2, an RNA virus, is mainly detected by RLRs through the RIG-1/MAVS pathway [14]. There is increasing evidence supporting the role of the cGAS/STING pathway, a DNA sensor [15], on the pathogenesis of SARS-CoV-2 infection and progression of COVID-19. It is recognized that SARS-CoV-2 infection has a dual impact on STING signaling, dependent on the disease stage and the affected tissues. In the initial stages of the disease, several SARS-CoV-2 proteins can suppress STING signaling and reduce interferon production [16,17,18,19], enabling the virus to evade the innate immune response [20]. Surprisingly, studies indicate in moderate and severe cases, SARS-CoV-2 can enhance STING signaling within a few days after diagnosis [21], leading to increased IFN production and aberrant inflammatory response. Thus, STING inhibition/activation appears to be closely linked to the stage and severity of COVID-19.

This review examines the dual role of STING signaling in COVID-19 patients, with a particular emphasis on its potential therapeutic implications for disease management.

## 2. Materials and Methods

We conducted a search on PubMed using the keywords “COVID-19”, “STING”, and “innate immune response” from the database’s inception to 13 February 2023 for studies published in English.

A total of 35 articles were retrieved. Titles, abstracts, and full texts were independently screened by three reviewers (D.F., J.L., and M.B.). Studies discussing rare comorbidities and specific virus variants were excluded. This screening process ensured that the subsequent analysis focused on data directly relevant to understanding the immunological mechanisms and potential therapeutic approaches associated with COVID-19.

## 3. Results

Of the thirty-four articles found, according to the search terms, two were excluded as they focused on rare comorbidities or specific virus variants. The remaining thirty-two studies included in the review are summarized in Tables S1 and S2, categorized by study design and their key findings. Among them, twelve are literature reviews primarily exploring how the cGAS/STING pathway influences the progression and severity of COVID-19, with potential therapeutic implications [20,22,23,24,25,26,27,28,29,30,31,32] (Table S1).

Regarding experimental research, two are clinical studies [33,34], along with two in vivo studies conducted in mice [35,36], and twelve in vitro studies using cell cultures [16,17,18,37,38,39,40,41,42,43,44]. Additionally, four studies integrated multiple methodologies. One study combined clinical (analyzing skin and lung lesions from COVID-19 patients), in vivo (mice), and in vitro (cell culture) approaches [45]. Another study incorporated both clinical and in vitro (cell culture) designs [46], while two other studies merged in vivo (mice or a combination of mice and zebrafish) and in vitro (cell culture) methodologies [47,48] (Table S2).

## 4. Discussion

**Figure 1:**
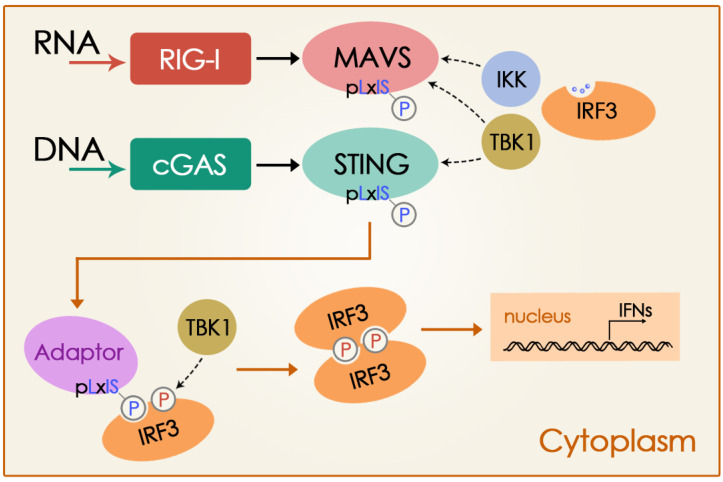
Innate immune response activation by RNA and DNA sensors. MAVS and STING are activated by viral RNA or cytosolic DNA, activating kinases IKK and TBK1. These, in turn, phosphorylate the adaptor proteins (MAVS or STING), which recruit IRF3, allowing its phosphorylation by TBK1. Phosphorylated IRF3 suffers dimerization and induces IFN in the nucleus. Non-continuous arrows indicate recruitment and activation. Modified from Liu et al. [12]. Reproduced with permission.

Pathogenic nucleic acids originating from invading microbes or damaged cellular organelles are strong triggers of the innate immune defenses for which the production of IFN is particularly important [49]. In the cytoplasm, RNA viruses are detected in the cytosol by RIG-I-like sensors, while microbial DNA or self-DNA is recognized by cGAS [6,50]. To initiate IFN production after RNA or DNA binding, these receptors must phosphorylate innate immune adaptor proteins (MAVS and STING, respectively) [12]. Interaction of RNA with RGI-like receptor activates MAVS, which in turn recruits and activates kinases IKK and TBK1 that then phosphorylate MAVS. This adaptor protein, once phosphorylated, recruits the transcription factor IRF3, enabling its phosphorylation by TBK1. Phosphorylated IRF3 then dissociates from the adaptor protein, dimerizes, and translocates to the nucleus to induce IFNs [12,14]. cGAS, a cytoplasmic DNA sensor [6], and its adaptor protein, STING, share a similar mechanism to induce IFNs [12]. Upon binding to DNA, cGAS is activated and catalyzes the production of cyclic GMP–AMP (cGAMP) from GTP and ATP [7]. As a secondary messenger, cGAMP binds to STING, leading to its activation [5,7,8,9,15,51,52]. STING then recruits and activates TBK1 (but not IKK), which then phosphorylates STING. This adaptor protein, once phosphorylated, recruits the transcription factor IRF3, which is then phosphoryled by TBK1, dissociates from STING, dimerizes, and translocates to the nucleus to induce IFNs and other cytokines [12,53]. Furthermore, STING serves as a key node in the activation of NF-kB and autophagy [22]. It is important to highlight that STING also plays a role in viral RNA sensing by interacting with MAVS [5,24] (Figure 1).

The observed parallels between T and B cell responses in COVID-19 and those found in both animal and human models with excessive STING activation—such as STING-associated vasculopathy with onset in infancy (SAVI syndrome)—have prompted investigations into the role of STING in COVID-19 pathogenesis [22]. Although SARS-CoV-2 is an RNA virus and the cGAS-STING pathway is primarily involved in DNA virus detection [5,15], studies have shown that it also plays a notable role in host innate immunity against certain single-stranded RNA viruses with no DNA intermediates in their life cycle [19]. Co-immunoprecipitation data suggest that STING may contribute to RNA virus sensing by interacting with the RNA sensor RIG-I and its adaptor protein MAVS [5]. More recently, it was shown that cytoplasmic chromatin sensing by cGAS is responsible for triggering the innate immune response in SARS-CoV-2 infection. The infection elevates cellular levels of cGAMP, which correlates with STING activation. cGAS detects chromatin DNA that is transported from the nucleus, a process driven by cell-to-cell fusion events during SARS-CoV-2 infection. Additionally, it has been shown that the cytoplasmic chromatin-cGAS-STING axis—not the MAVS-dependent viral RNA sensing pathway—plays a key role in inducing interferon and pro-inflammatory gene expression following cell fusion [43]. This mechanism is consistent with the dual role played by the cGAS-STING pathway in distinct stages and severities of COVID-19. In the early stages, SARS-CoV-2 proteins inhibit the STING pathway, hindering the antiviral response and promoting it. In later stages, the fusion of ACE2 and viral S proteins leads to the formation of syncytia, which contains micronuclei (MN). This results in DNA damage and subsequent activation of the cGAS-STING pathway, ultimately resulting in increased IFN expression (Figure 1). Constant activation of DNA signaling through cGAS-STING provokes dysregulated immune responses, leading to excessive inflammation, tissue damage, and worsening clinical outcomes [41]. The formation of syncytia provides a plausible explanation for late IFN response in moderate/severe COVID-19 patients [20]. Neufeldt et al. [54] showed that activation of the cGAS-STING axis in response to cellular DNA damaged by the fusion of ACE2 and viral S protein directly activates NF-κβ, resulting in an inflammatory response due to the blockage of other immune pathways. It has been suggested that RNA viruses contribute to chromosomal instability and the formation of MN. Individuals with elevated levels of lymphocyte MN tend to have a compromised immune response, making them more vulnerable to RNA virus infections. Additionally, the leakage of DNA from the MN and viral RNA can synergistically amplify cytokine production through the cGAS-STING pathway [26].

AMPK is the main sensor of glucose deficiency, overseeing multiple metabolic pathways. Recent studies have also identified AMPK as a STING activator. In the early stages of viral infection, a sharp drop in blood glucose levels leads to strong AMPK activation. This activation occurs at the lysosomal membrane mediated by aldolase and the v-ATPase-Regulator complex. It is also influenced by the AMP/ATP ratios and interaction with other signaling pathways. Once activated, AMPK phosphorylates TBK1 at S511, which recruits IRF3, promoting the assembly of STING and MAVS signalosomes, thereby strengthening the immune system’s ability to detect pathogens and cellular damage. In this way, AMPK-mediated TBK1 phosphorylation links glucose availability to the intensity of the innate immune response. This metabolic–immune interaction may help explain the strong correlation between diabetes, poor glycemic control, and the increased severity and mortality risk in COVID-19 patients. In response to early viral infection, AMPK activation plays a pivotal role in shaping the magnitude of the innate antiviral response [47]. Given its role in immune regulation, it has been proposed that targeting AMPK pharmacologically, a feasible strategy, may provide therapeutic advantages for infectious disease treatment [34]. On the other hand, the activation of cGAMP synthase does not appear to be linked to lung fibrosis-like changes observed in post-COVID-19 (PC) patients. Instead, this role is attributed to the absence of melanoma-2 (AIM2) inflammasome. Peripheral blood mononuclear cells (PBMCs) derived from PC patients without signs of lung fibrosis showed no AIM2 activation. In contrast, PBMCs from PC patients who exhibited lung fibrosis markers displayed heightened AIM2 activation, leading to the release of IL-1α, IFN-α, and TGF-β [33].

Human endogenous retroviruses (HERVs) are remnants of ancient viral integrations that have become part of the human genome through Mendelian inheritance and evolutionary processes. These elements now constitute approximately 8% of the human genome. Among them, HERV-K (HML-2) is the most transcriptionally active subtype, playing a key role in embryogenesis. Increased interferon (IFN) production in COVID-19 patients has been linked to HERV-K (HML-2) activation, suggesting a potentially beneficial effect in individuals affected by the disease [34].

One of the immune evasion strategies employed by SARS-CoV-2 involves suppressing type I and III IFN responses, a phenomenon observed in severe cases. Several viral proteins interfere with the cGAS-STING pathway, disrupting autophagy and IFN production, thereby promoting viral replication and increasing disease severity [17,18,30,42,54]. ORF3a was shown to be a selective inhibitor of STING-triggered autophagy, facilitating viral replication [16,42]. Interestingly, this inhibitory ability seems to be an acquired trait of SARS-CoV-2 ORF 3a, as the ORF3 protein from the original SARS-CoV strain does not exhibit the same inhibitory effect [42]. Inhibition of the c-GAS-STING pathway results in NF-kB accumulation and excessive cytokine release, leading to an overactive inflammatory response [16]. Additionally, SARS-CoV-2 ORF9b disrupts type I and III IFN activation by targeting multiple components of the cGAS-STING signaling network. This ORF interacts with MDA-5, RIG-I, STING, MAVS, and TBK1, thereby preventing IRF3 phosphorylation and nuclear translocation [17,30]. Another viral protein, ORF10, binds to TBK1 kinase, impairing this signaling cascade, leading to decreased IFN production and autophagy suppression, which is essential for the degradation of cellular components and antiviral defense. These actions of ORF10 contribute to SARS-CoV-2 immune evasion, facilitating infection persistence and disease progression [18]. The SARS-CoV-2 main protease, 3CLpro, has also been shown to suppress immune responses mediated by the RLR and cGAS-STING pathways [16,30]. Another key viral protein, nonstructural protein 7 (NSP7), interacts with cytosolic RNA sensors RIG-I and MDA5, impairing RIG-I/MDA5 signalosome formation and STING signal transduction, which ultimately reduces IFN production [37]. Host factors also modulate the cGAS-STING pathway, presenting therapeutic potential. In human intestinal epithelial cells, USP22 has been identified as a critical regulator of type III IFN signaling, acting through STING pathway activation to protect against viral infections [38].

Furthermore, in severe cases of COVID-19, elevated levels of IL-1β and IL-6 cytokines have been observed. Studies indicate that while primary human airway epithelial (HAE) cells possess functional inflammasomes supporting SARS-CoV-2 replication, they do not serve as the primary source of IL-1β secretion during infection. Instead, SARS-CoV-2-infected HAE cells generate a secondary signal, including mitochondrial and genomic DNA, that triggers leukocyte-derived IL-1β release. Once secreted, IL-1β further stimulates IL-6 production in HAE cells. Notably, STING has been found to suppress leukocyte IL-1β secretion, suggesting a potential role in regulating the inflammatory response [46].

**Figure 2:**
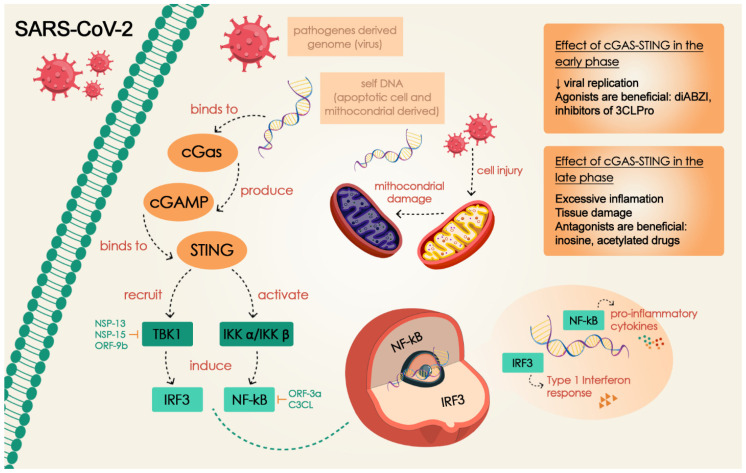
The dual role of cGAS-STING signaling in COVID-19 and potential therapeutic targeting. In the early phase of infection, the virus can suppress cGAS-STING signaling to evade immune detection. Thus, cGAS-STING agonists are beneficial at this stage in order to reduce viral replication and control infection. However, in the late stages, activation of this pathway can lead to excessive inflammation and tissue damage, exacerbating disease severity. In this case, therapy might antagonize cGAS-STING. Modified from Elahi et al. Downward arrow indicates reduction [55].

Thus, the cGAS-STING pathway plays a crucial role in the progression of COVID-19. Early recognition of viral RNA by PRRs benefits the host by triggering type I IFN production and providing antiviral defense. However, delayed activation of the cGAS-STING pathway contributes to NF-kB accumulation, hyperinflammatory response, and tissue damage [45]. This knowledge may also be employed in the therapy of COVID-19. STING agonists may be employed early in infection to enhance the antiviral response [40,48], while STING inhibitors have been shown to reduce severe lung inflammation caused by SARS-CoV-2, thereby improving disease outcomes [20,45] (Figure 2). Additionally, intranasal administration of SARS-CoV-2 spike proteins (S-NPs) combined with cGAMP has been shown to robustly stimulate antibody responses in the respiratory tract of mice. This approach enhanced IgA and IgG antibody production, targeting spike proteins in bronchoalveolar lavages and lung tissues. The induced antibodies effectively neutralized both the wild-type and Delta variant strains of SARS-CoV-2. Moreover, intranasal immunization also triggered systemic immune responses, further increasing IgA and IgG production. These findings suggest that cGAMP-based nasal formulations could enhance the efficacy of SARS-CoV-2 vaccines [35].

During the early phase of COVID-19, controlling viral replication and enhancing the IFN response through immune-modulating drugs may be a viable strategy to keep the immune balance and prevent disease progression [43,44]. In this context, the STING agonist dimeric amidobenzimidazole (diABZI) has shown promise in inhibiting SARS-CoV-2 replication by inducing transient IFN signaling activation and significantly reducing viral replication. This compound has demonstrated potent anti-coronavirus activity in both cell culture systems and mouse models, with minimal cytotoxic effects [43,44,48]. Moreover, low-dose treatment of diABZI (0.1 μM) effectively lowered the SARS-CoV-2 viral load at the epithelial apical surface, preventing damage to epithelial cells in a reconstituted primary human bronchial airway epithelial system [44]. The innate immune effector IRF3, which operates downstream of STING, plays a crucial role in this antiviral mechanism [40]. SARS-CoV-2 papain-like (PLpro) enzyme facilitates viral replication while modulating host immune responses by interfering with key signaling pathways. This enzyme plays a crucial role in viral replication and suppression of type I IFN activation through multiple mechanisms, including (a) inhibiting STING dimerization, (b) disrupting the formation of the MAVS-STING-RIG-I complex, (c) deISGylating ISG15, (d) deregulating MAPK, TGF-β, and NF-κB pathways, (e) deubiquitinating STING, RIG-I, TBK1, and IRF3, and (f) preventing TBK1 phosphorylation by deubiquitinating TRAF3 and TRAF6. Several PLpro inhibitors have been identified, including FDA-approved drugs, which can suppress viral replication while enhancing the host’s innate immune response [29].

Conversely, in severe or late-stage COVID-19, cGAS-STING pathway antagonists may offer therapeutic benefits. In SARS-CoV-2-infected mice, inosine was shown to counteract IL-6 overexpression, mitigating acute inflammatory lung injury and improving survival. Additionally, inosine inhibited TBK1 phosphorylation by binding to STING and glycogen synthase kinase-3β (GSK3β), thereby suppressing pro-inflammatory IL-6 while promoting anti-inflammatory IL-10 [36]. Repurposing existing drugs has also been proposed as a potential strategy against COVID-19. Acetylated derivatives of aspirin and dapsone have been shown to acetylate cGAS, thereby inhibiting cGAS-mediated signaling. This inhibition helps regulate cGAS activity, reducing IFN-I production and NF-κB signaling through the STING pathway, ultimately contributing to immune control and inflammation reduction [39].

It is important to emphasize that cGAS-STING pathway activation may influence vaccine efficacy, as cGAMP is utilized as an adjuvant in intranasal COVID-19 vaccines to enhance the immune response [35]. Additionally, certain anti-inflammatory drugs and immune response inhibitors, including IL-6 inhibitors and corticosteroids, can interact with the cGAS-STING pathway by reducing STING-mediated cytokine production and inflammatory responses [25].

Despite advancements in understanding the role of cGAS-STING signaling in COVID-19 and its potential therapeutic applications, several challenges remain. The dual nature of this pathway in disease progression—promoting an antiviral innate response while, when excessively activated, leading to chronic inflammation and tissue damage—complicates its therapeutic modulation. The factors that regulate this delicate balance are not yet fully elucidated, making targeted therapeutic interventions challenging. Furthermore, variations in cGAS-STING pathway activation in response to different SARS-CoV-2 strains add another layer of complexity. The immune response also varies according to host-specific factors, such as genetic background, age, and comorbidities, making it difficult to extrapolate clinical data for a comprehensive understanding of the molecular mechanisms underlying COVID-19 pathogenesis [45]. Although potential therapies for modulating the cGAS-STING pathway exist, there is no consensus on the optimal timing or patient profile for their use. Moreover, translating experimental findings into clinical practice is hindered by the lack of appropriate experimental designs [30]. Another significant challenge is the difficulty in obtaining biological samples due to ethical and logistical constraints. During the pandemic, patient participation in voluntary studies was not always feasible, which limited the development of biobanks and the validation of experimental findings [56].

## 5. Conclusions

Grasping the interactions among viral signaling pathways, host immune defenses, and regulatory processes is essential for developing effective therapeutic strategies against COVID-19. The cGAS-STING signalosome exhibits a dual role during SARS-CoV-2 infection. During the early phase of infection, the virus suppresses cGAS-STING signaling to evade immune detection. Several viral proteins are implicated in this process, such as ORF3a, ORF9b, ORF10, nsp7, and 3CLPro. Targeting them may be an attractive approach to reduce viral replication and enhance immune control. However, in later stages of the disease, cGAS-STING pathway activation can trigger excessive inflammation and tissue damage, worsening disease severity. Therapeutic modulation of this pathway—whether through agonists like dimeric amidobenzimidazole (diABZI) to boost early immune responses or inhibitors targeting viral proteins such as 3CLpro to prevent hyperinflammation—offers a promising personalized approach. These interventions could help balance immune responses, mitigate severe inflammation, and ultimately improve clinical outcomes in patients with severe COVID-19.

## Data Availability

No new data were created that should be made available.

## Supplementary Materials

The following supporting information can be downloaded at https://www.mdpi.com/article/10.3390/cells14050362/s1. Table S1. Review studies evaluating the role of STING signaling in the pathogenesis and therapy of COVID-19. Table S2. Clinical and experimental studies evaluating the role of STING signaling in the pathogenesis and therapy of COVID-19.
