# Supplementary material for: The Dual Role of cGAS-STING Signaling in COVID-19: Implications for Therapy
Source: Cells. 2025 Feb 28;14(5):362. doi: 10.3390/cells14050362 (PMC11899623; doi:10.3390/cells14050362)
Supplement: Supplementary file 1 [file cells-14-00362-s001.zip › cells-3409396-supplementary.pdf]

Table S1. Review studies evaluating the role of STING signaling in the pathogenesis and therapy of COVID-19

Author/date	Title	Main Results
Anwar et al. (2021) [24]	cGAS-STING-mediated sensing pathways in DNA and RNA virus infections: crosstalk with other sensing pathways	The detection of viral genomes or their replication intermediates within host cells is carried out by cytosolic proteins. cGAS and IFI16 identify foreign DNA, while RIG-I and MDA5 detect foreign RNA. Activation of these sensors initiates a cascade of signaling events that activate downstream molecules, ultimately leading to the transcription of type I and III IFNs. These IFNsinterferons are crucial in curbing viral spread, either by directly inhibiting viral replication or by prompting host cells to block viral protein synthesis. The antiviral immune response primarily depends on the recognition of viral genomes and subsequent signaling. Although distinct receptor proteins detect DNA and RNA viruses, there is potential for overlap between the mechanisms for sensing viral DNA and RNA.
Berthelot et al. (2020) [22]	Lymphocyte Changes in Severe COVID-19: Delayed Over-Activation of STING?	There are similarities between T and B cells responses in COVID-19 and animal and human models involving STING activation (STING-associated vasculopathy with onset in infancy – SAVI syndrome). These similarities may be clues for late activation on STING in severe COVID-19 patients. The overactivation of the STING pathway results in a significant increase in the production of IL-6 and other cytokines, leading to hyper-inflammatory responses and lymphopenia. CD4+ and CD8+ T cells are particularly affected by the late activation of the STING pathway, contributing to the severity of the disease.
Carty et al. (2021) [23]	Detection of Viral Infections by Innate Immunity	PRRs activate signaling pathways that result in the expression of pro-inflammatory cytokines and type I and II IFNs, which in turn induce interferon stimulated genes (ISG) that help establish an antiviral state. Inflammasomes promote antiviral responses by releasing IL-1 and IL-18 and inducing pyroptotic cell death. Exacerbated innate immune responses can contribute to viral pathology.
Copaescu et al. (2020) [25]	The role of IL-6 and other mediators in the cytokine storm associated with SARS-CoV-2 infection	Dysregulated immune responses have been observed in a smaller subset of individuals infected with SARS-CoV-2, leading to clinical deterioration typically 7 to 10 days after the initial presentation. This hyperinflammatory state, often referred to as a cytokine storm in its most severe form, is characterized by elevated levels of IL-6, IL-10, TNF-α, and other cytokines, along with severe CD4+ and CD8+ T-cell lymphopenia and coagulopathy. Identifying patients at risk could enable the early implementation of aggressive intensive care, antiviral therapy, and immunomodulatory treatments to mitigate complications associated with this proinflammatory state. Several reports and ongoing clinical trials offer hope that available immunomodulatory therapies may have therapeutic potential in these severe cases.
Ji et al. (2022) [27]	E3 Ubiquitin Ligases: The Operators of the Ubiquitin Code That Regulates the RLR and cGAS-STING Pathways	Ubiquitin E3 ligases (E3s) are the direct manipulator of ubiquitin codons and determine the type and modification type of substrate proteins. Therefore, members of the E3s family are involved in balancing the host's innate antiviral immune responses. E3s modulate the activities of RLRs and the cGAS-STING pathway, which are fundamental in detecting viral RNA and DNA, respectively. The activation of these pathways leads to the production of type I IFNs and other inflammatory cytokines, essential for antiviral defense. Dysregulation of these ligases can result in deficiencies in antiviral defense or excessive inflammation.
Kirsch-Volders et al (2021) [26]	Inflammatory cytokine storms severity may be fueled by interactions of micronuclei and RNA viruses such as COVID-19 virus SARS-CoV-2. A hypothesis	Individuals with high levels of lymphocytes containing micronuclei (MN) are more susceptible to RNA virus infections and exhibit more intense inflammatory responses. The leakage of DNA from the MN and viral RNA can synergistically amplify cytokine production through the cGAS-STING pathway, suggesting potential therapeutic strategies for controlling viral infections and excessive inflammation

Mahmoudvand & Shokri (2021) [29]	Interactions between SARS coronavirus 2 papain-like protease and immune system: A potential drug target for the treatment of COVID-19	SARS-CoV-2 papain-like (PLpro) enzyme facilitates viral replication and modulates host immune responses by interacting with critical signaling pathways such as STING and NF-κB. The enzyme plays a crucial role in viral replication and inhibits the activation of type I IFN by: (a) reducing STING dimerization, (b) disrupting the formation of the MAVS-STING-RIG-I complex, (c) deISGylating ISG15, (d) deregulating TGF-β, MAPK, and NF-κB pathways, (e) deubiquitinating RIG-I, STING, IRF3, and TBK1, and (f) preventing TBK1 phosphorylation by deubiquitinating TRAF3 and TRAF6. Several PLpro inhibitors have been identified, many of them are FDA-approved. They can interfere with the host's innate immune system, suppress viral replication, and enhance immune responses. This establishes a dual therapeutic strategy against COVID-19.
Mdkhana et al. (2021) [31]	Nucleic Acid-Sensing Pathways During SARS-CoV-2 Infection: Expectations versus Reality	Balance between inflammation and antiviral response is crucial for the pathogenesis of SARS-CoV-2. SARS-CoV-2 infection and evasion mechanisms activate NOD-like receptor (NLR) signaling and the NLRP3 pathway. This activation results in the production of inflammatory cytokines (IL-1β and IL-6), while muting or blocking cGAS-STING and interferon type I and III pathways, resulting in decreased production of antiviral interferons and delayed innate response. Therapeutic strategy aims to block the inflammatory pathway while simultaneously stimulating the production of IFN. This control might be able to provide early control of viral replication and dissemination, prevent disease progression, and cytokine storm development.
de Moura Rodrigues et al. (2022) [28]	STING Targeting in Lung Diseases	The STING pathway offers a promising route for the treatment of pulmonary diseases with currently limited therapeutic options. The ability to modulate this pathway can significantly improve patient outcomes by enhancing the immune response, reducing inflammation, and mitigating fibrosis.
Xiao & Zhang (2022) [20]	Involvement of the STING signaling in COVID-19	In the early stages of SARS-CoV-2 infection, viral proteins inhibit the activation of the STING pathway and prevent the antiviral response, facilitating viral replication. In the later phase, ACE2 and the viral S proteins fuse, forming syncytia that contain micronuclei and mediate DNA damage and activate the STING pathway. Prolonged activation of the STING pathway results in an abnormal inflammatory response, causing tissue damage and a poor prognosis. STING agonists can be used to activate the STING pathway at the onset of infection, promoting an antiviral response. On the other hand, STING inhibitors can be used to mitigate tissue damage by suppressing excessive STING activation and the abnormal inflammatory response.
Wu et al. (2022) [30]	Progress of cGAS-STING signaling in response to SARS-CoV-2 infection	SARS-CoV-2 proteins ORF9a and ORF3 inhibit the production of type I and III IFNs by targeting constituents of the cGAS-STING signaling pathways. SARS-CoV-2 nsp5, nsp6, nsp13 and N protein<br>were reported to inhibit the phosphorylation of TBK1. SARS-CoV-2 protease (3CLpro) inhibits STING by destroying the assembly of the STING complex and downstream signal transduction. SARS-CoV-2 nsp5, nsp6, nsp13 and N protein inhibit the phosphorylation of TBK1.<br>STING agonists, such as diABZI, exert a beneficial effect by transiently stimulating IFN signaling triggered by cGAS-STING after detection of SARS-CoV-2 infection.
Yan et al. (2021) [32]	Spatial and temporal roles of SARS-CoV PLpro—A snapshot	SARS-CoV PLpro plays multifaceted roles in viral replication and immune modulation. Understanding these roles can help in developing targeted therapies to mitigate SARS-CoV infections.

Table S2. Clinical and experimental studies evaluating the role of STING signaling in the pathogenesis and therapy of COVID-19

Author/date	Title	Study design	Main Results
Barnett et al. (2023) [46]	An epithelial-immune circuit amplifies inflammasome and IL-6 responses to SARS-CoV-2	Clinical and Experimental research (in vitro; cells)	Elevated levels of the cytokines IL-1β and IL-6 are associated with severe COVID-19. While primary human airway epithelia (HAE) have functional inflammasomes and support SARS-CoV-2 replication, they are not the source of IL-1β released upon infection. In leukocytes, the SARS-CoV-2 E protein upregulates inflammasome gene transcription via TLR2 to prime, but not activate, inflammasomes. SARS-CoV-2-infected HAE supply a second signal, which includes genomic and mitochondrial DNA, to stimulate leukocyte IL-1 β release. Nuclease treatment, STING, and caspase-1 inhibition but not NLRP3 inhibition blocked leukocyte IL-1β release. After release, IL-1β stimulates IL-6 secretion from HAE. Therefore, infection alone does not increase IL-1β secretion by either cell type. Rather, bi-directional interactions between the SARS-CoV-2-infected epithelium and immune bystanders stimulates both IL-1β and IL-6, creating a pro-inflammatory cytokine circuit. Consistent with these observations, patient autopsy lungs show elevated myeloid inflammasome gene signatures in severe COVID-19.
Colarusso et al. (2022) [33]	Activation of the AIM2 Receptor in Circulating Cells of Post-COVID-19 Patients With Signs of Lung Fibrosis Is Associated With the Release of IL-1α, IFN-α and TGF-β	Clinical research	The study evaluated the role of the Absent in melanoma-2 (AIM2) inflammasome in Post-COVID-19 (PC)-associated lung fibrosis-like changes revealed by chest CT scans. Peripheral blood mononuclear cells (PBMCs) obtained from PC patients who did not develop signs of lung fibrosis were not responsive to AIM2 activation by Poly dA:dT. In contrast, PBMCs from PC patients with signs of lung fibrosis were highly responsive to AIM2 activation, which induced the release of IL-1 α, IFN- α and TGF-β. The recognition of Poly dA:dT was not due to the activation of cyclic GMP-AMP (cGAMP) synthase, a stimulator of interferon response (cGAS-STING) pathways, implying a role for AIM2 in PC conditions.
Deng et al. (2023) [37]	SARS-CoV-2 NSP7 inhibits type I and III IFN production by targeting the RIG-I/MDA5, TRIF, and STING signaling pathways	Experimental research (in vitro; cells)	SARS-CoV-2 nonstructural protein 7 (NSP7) inhibits the expression of type I and type III IFNs by interfering with RIG-I/MDA5−MAVS signalosome formation and STING signaling transduction. The study provides the first evidence that SARS-CoV-2 NSP7 interacts with the cytosolic RNA sensors RIG-I and MDA5 and represses RIG-I/MDA5 signaling pathway-induced IFN production.
Domizio et al. (2022) [45]	The cGAS–STING pathway drives type I IFN immunopathology in COVID-19	Clinical (skin and lung lesions of COVID-19 patients) and experimental research (in	cGAS-STING pathway is a critical driver of aberrant type I IFN responses in COVID-19. Early detection of viral RNA by PRRs can be beneficial for the host, with activation of type I IFN and antiviral protection. Late activation of the cGAS-STING pathway promotes NF-kB accumulation, hyperinflammatory response and tissue damage. Pharmacological inhibition of STING reduces severe lung inflammation induced by SARS-CoV-2 and improves disease

		vivo: mice and in vitro: cells)	outcome.
Guo et al. (2022) [34]	High Expression of HERV-K (HML-2) Might Stimulate Interferon in COVID-19 Patients	Clinical research	HERV-K (HML-2) gag, env, and pol genes were highly expressed in COVID-19 patients and VERO cells infected with SARS-CoV-2. The interferon-related genes IFNB1, ISG15, and IFIT1 were also activated in COVID-19 patients. GO analysis showed that HERV-K (HML-2) can regulate the secretion of interferon. The high expression of HERV-K (HML-2) might activate the increase of interferon through the cGAS-STING pathway in COVID-19 patients, proving that HERV-K might have a beneficial role.
Han et al. (2022) [18]	SARS-CoV-2 ORF10 antagonizes STING-dependent interferon activation and autophagy	Experimental research (in vitro; cells)	The ORF10 protein interacts with the TBK1 kinase protein, a crucial component in the cGAS-STING signaling pathway, responsible for the activation of antiviral immune responses. By interacting with TBK1, ORF10 inhibits the activation of this signaling pathway, resulting in a decrease in interferon production and the autophagy process, which is essential for the degradation of cellular components and antiviral defense. These actions of ORF10 contribute to the immune evasion of SARS-CoV-2, facilitating infection and disease progression. The interaction between ORF10 and TBK1 paves the way for new therapeutic strategies.
Han et al. (2021) [17]	SARS-CoV-2 ORF9b antagonizes type I and III interferons by targeting multiple components of the RIG-I/MDA-5-MAVS, TLR3-TRIF, and cGAS-STING signaling pathways	Experimental research (in vitro; cells)	SARS-CoV-2 ORF9b inhibited the activation of types I and III IFNs induced by the components of cytosolic dsRNA-sensing pathways of RIG-I/MDA5-MAVS signaling, including RIG-I, MDA-5, MAVS, TBK1, and IKKε. SARS-CoV-2 ORF9b also suppressed the induction of types I and III IFNs by STING. A mechanistic analysis revealed that the SARS-CoV-2 ORF9b protein interacted with RIG-I, MDA-5, MAVS, STING, and TBK1 and impeded the phosphorylation and nuclear translocation of IRF3. SARS-CoV-2 ORF9b negatively regulates antiviral immunity and thus facilitates viral replication.
Jearanaiwitayakul et al., (2022) [35]	The STING Ligand and Delivery System Synergistically Enhance the Immunogenicity of an Intranasal Spike SARS-CoV-2 Vaccine Candidate	Experimental research (in vivo; mice)	cGAMP is a STING ligand, playing a role in the activation of the IRF-3 and NF-kB immune pathways. This activation results in the release of pro-inflammatory cytokines and type I interferon (IFN), which are crucial for the recruitment of type 1 helper T cells (Th-1). The intranasal administration of SARS-CoV-2 spike proteins (S-NPs) along with cGAMP conferred a robust stimulation of antibody responses in the respiratory tract of mice, leading to an increase of IgA and IgG antibodies toward the spike proteins in bronchoalveolar lavages and the lungs. The elicited antibodies were able to neutralize both the wild-type and Delta variant strains of SARS-CoV-2. The intranasal immunization also stimulated systemic responses, increasing the production of IgA and IgG. Thus, the use of c-GAMP in nasal formulations can enhance the efficacy of vaccines, especially against SARS-CoV-2.
Karlowitz et al. (2022) [38]	USP22 controls type III interferon signaling and SARS-CoV-2 infection through activation of STING	Experimental research (in vitro; cells)	Host factor USP22 plays a crucial role in regulating type III IFN signaling and defending against viral infections in human intestinal epithelial cells. The activation of the STING pathway mediated by USP22 offers a robust antiviral defense against SARS-CoV-2. Modulating USP22 presents itself as a promising therapeutic target for combating viral infections
Lee et al. (2022) [39]	COVID-19 Molecular Pathophysiology: Acetylation of	Experimental research (in vitro; cells)	The use of existing medications, as well as modification of currently available drugs through acetylation, for example, have been proposed as strategies to combat COVID-19. Aspirin and Dapsone can

	Repurposing Drugs		acetylate cGAS, thus inhibiting cGAS-mediated signaling. This contributes to the control of cGAS activity, reducing the production of IFN-1 and NF-κB signaling via STING. These approaches could be employed for the development of effective therapies against COVID-19.
Li et al. (2022) [48]	Pharmacological activation of STING blocks SARS-CoV-2 infection	Experimental research (in vivo: mice and in vitro: cells)	The STING agonist diABZI effectively inhibited SARS-CoV-2 infection across various strains, including variants of concern like B.1.351, by transiently enhancing IFN signaling. Notably, diABZI limits viral replication in primary human bronchial epithelial cells and in vivo in mice.
Liu et al. (2021) [40]	Activation of STING Signaling Pathway Effectively Blocks Human Coronavirus Infection	Experimental research (in vitro; cells)	HCoV-OC43 infection did not stimulate the STING signaling pathway, but the activation of STING signaling effectively inhibits HCoV-OC43 infection to a much greater extent than that of type I interferons (IFNs). IRF3, the key STING downstream innate immune effector, is essential for this anticoronavirus activity. diABZI robustly blocks the infection of not only HCoV-OC43 but also SARS-CoV-2. This study identifies the STING signaling pathway as a potential therapeutic target that could be exploited for developing broad-spectrum antiviral therapeutics against multiple coronavirus
Ren et al. (2021) [41]	Micronucleus production, activation of DNA damage response and cGAS-STING signaling in syncytia induced by SARS-CoV-2 infection	Experimental research (in vitro; cells)	Fusion of the viral S protein with ACE2 leads to the formation of syncytia, leading to the production of micronuclei. This results in DNA damage, which activates the cGAS-STING pathway. Activation of cGAS-STING leads to upregulation of IFN expression. Constant activation of DNA signaling and cGAS-STING provokes aberrant immune activation, which leads to tissue damage found in severe and advanced stage COVID-19 patients.
Rui et al. (2020) [16]	Unique and complementary suppression of cGAS-STING and RNA sensing- triggered innate immune responses by SARS-CoV-2 proteins	Experimental research (in vitro; cells)	SARS-CoV-2 structural proteins, accessory proteins and the main viral protease (3CL) are potent inhibitors of host innate immune responses of distinct pathways. The fusion of the virus's S protein with the ACE2 receptor on the host cell causes mitochondrial damage and activates the cGAS-STING signaling pathway. ORF3a inhibits STING, but not RLR response. Viral protease 3CL was a potent inhibitor of both the RLR and cGAS-STING pathways.
Su et al. (2023) [42]	SARS-CoV-2 ORF3a inhibits cGAS-STING-mediated autophagy flux and antiviral function	Experimental research (in vitro; cells)	The ORF3a protein of SARS-CoV-2 is a key component in the pathogenesis of COVID-19 due to its ability to inhibit the cGAS-STING autophagy pathway but not IRF3-type I IFN induction. This novel function of ORF3a, distinct from targeting autophagosome-lysosome fusion, is a selective inhibition of STING-triggered autophagy to facilitate viral replication. The ability to inhibit STING induced autophagy appears to be an acquired function of SARS-CoV-2 ORF 3a, since SARS-CoV ORF3 lacks this function. Targeting the inhibition of ORF3a represents a strategy for developing therapies that can enhance the host's antiviral defenses, potentially mitigating the severity of the infection.
Wang et al. (2023) [36]	Inosine: A broad-spectrum anti-	Experimental research (in	Inosine abrogated IL-6 overexpression to ameliorate acute inflammatory lung injury induced by SARS-CoV-2 infection. In

	inflammatory against SARS-CoV-2 infection-induced acute lung injury via suppressing TBK1 phosphorylation	vivo; mice)	addition, inosine inhibited the phosphorylation of TBK1 through binding to STING and glycogen synthase kinase-3b (GSK3β), downregulating proinflammatory IL-6 and upregulating anti-inflammatory IL-10, thus ameliorating acute inflammatory lung injury and improving survival in mice infected with SARS-CoV-2.
Zhang et al. (2022) [47]	AMPK directly phosphorylates TBK1 to integrate glucose sensing into innate immunity	Experimental research (in vivo: mice and zebrafish and in vitro; cells)	AMPK, a central enzyme in cellular energy control, is strongly activated in various tissues during the very early stage of viral infection due to a rapid and sharp reduction in blood glucose concentrations. Once activated, AMP directly phosphorylates TBK1, promoting the assembly of STING and MAVS signalosomes, thus potentiating pathogen and damage surveillance. AMPK thus plays a role at the intersection between glucose detection and innate immunity through the phosphorylation of TBK1. Pharmacological manipulation of AMPK presents significant therapeutic potential.
Zhou et al. (2021) [43]	Sensing of cytoplasmic chromatin by cGAS activates innate immune response in SARS-CoV-2 infection	Experimental research (in vitro; cells)	SARS-CoV-2 infection triggers the innate immune response via the cytosolic DNA sensing cGAS-STING pathway. The infection elevates cellular levels of cGAMP, which is linked to STING activation. cGAS detects chromatin DNA that is transported from the nucleus, a process driven by cell-to-cell fusion during SARS-CoV-2 infection. Furthermore, cytoplasmic chromatin-cGAS-STING pathway, but not MAVS-mediated viral RNA sensing pathway, contributes to interferon and pro-inflammatory gene expression upon cell fusion. cGAS is required for host antiviral responses against SARS-CoV-2, and diABZI, a STING-activating compound potently inhibits viral replica. The exploration of the syncytium-cGAS-STING pathway and the use of STING agonists like diABZI represent innovative approaches to stimulate IFN responses and contain the replication of SARS-CoV-2. These strategies can complement existing antiviral therapies and contribute to the control of COVID-19.
Zhu et al. (2021) [44]	Inhibition of coronavirus infection by a synthetic STING agonist in primary human airway system	Experimental research (in vitro; cells)	diABZI, a synthetic small molecule STING receptor agonist, showed potent anti-coronavirus activity against both the common cold human coronavirus 229E (HCoV-229E) and SARS-CoV-2 in cell culture systems, with minimum citotoxicity. The antiviral activity of diABZI was dependent on the interferon pathway in HCoV-229E infected normal human fibroblast lung cells (MRC-5) and reconstituted primary human airway air-liquid interface (ALI) cultures. Furthermore, low-dose of diABZI treatment at 0.1 μM effectively reduced the SARS-CoV-2 viral load at the epithelial apical surface and prevented epithelial damage in the reconstituted primary human bronchial airway epithelial ALI system. During the early stage of disease, suppressing viral replication and increasing the IFN response via immune modulator drugs, such as STING agonist, may be a viable approach to maintain a balanced immune response and prevent disease progression. diABZI has demonstrated comparable effectivity with Remdesevir with greater selectivity.
